# Commentary: 3D Laparoscopy-Assisted Operation to Adult Intussusceptions During Perioperative Period of Liver Transplantation: Case Report and Literature Review

**DOI:** 10.3389/fsurg.2021.764741

**Published:** 2021-10-20

**Authors:** Som P. Singh, Kiera G. Borthwick, Fahad M. Qureshi

**Affiliations:** ^1^Department of Biomedical Sciences, University of Missouri–Kansas City School of Medicine, Kansas, MO, United States; ^2^Department of Neurosciences, Washington and Lee University, Lexington, VA, United States

**Keywords:** 3D, laparoscopy, intussusceptions, liver transplant, outcomes

In the case report by Gao et al. (1), a 32-year-old male presented with pain and distention in the lower left quadrant following a liver transplant for liver failure and cirrhosis. Initial CT scans showed proximal enterostenosis, but symptoms of constipation gradually became more severe. Another CT scan was completed 4 days later, and an exploratory laparoscopy was performed, indicating antegrade intussusception, where the proximal bowel invaginated itself into the distal bowel. Adhesion removal and a simple reduction were completed, and a small incision was made to ensure local adhesion restoration. The serosa was sutured integrally. Reported pain and distention decreased gradually following the procedure, and the patient was discharged 9 days later, with no symptoms at a 2-month follow-up.

Laparoscopy is becoming a more favorable method for surgical treatment of intussusception and causes less tissue damage and immune responses compared to an open laparotomy (2). Moreover, laparoscopy decreases the duration of hospital stay and the time to first food intake (3). Post-operative complication rates are similar between laparotomy and laparoscopy, but the complications from laparoscopy are often more severe (i.e., perforation) (3).

Surgical options, including laparotomy or laparoscopy, remains the main treatment plan for adult intussusception, although the specifics of the interventions vary. Surgical interventions for intussusception tend to include one of the five following procedures: resection without reduction, reduction followed by resection, reduction only, enterotomy and mass excision, and negative exploration (4). Most studies support case-by-case determinations of whether reductions should be performed, while there is more limited support for resection without reduction. Resection without reduction helps prevent the potential risks of implantation metastasis in intussusception cases involving cancerous tumors. Overall, the best surgical method depends on the location of the intussusception. In enteric intussusception, reduction followed by resection is most successful. In colonic intussusception, resection alone is most effective. Ileocolic intussusception has an intermediate location and requires a more selective, combined approach (4).

There are also non-invasive and non-surgical treatments for intussusceptions, including hydrostatic, pneumatic, and gas enemas under ultrasound or fluoroscopy. In addition, colonoscopy has been considered as a treatment option. Unless there is an imminent indication for surgery, as in emergency cases, it is frequently recommended that diagnostic evaluation and more conservative, non-surgical treatment options be utilized, especially in pediatric populations (4, 5). Ultrasound-guided hydrostatic reductions can serve as a safer, non-operative treatment for intussusceptions (6). Hydrostatic reductions have similar safety and efficacy compared to pneumatic reduction, and ultrasound guided intussusception reduction should continue to be evaluated, as it does not require radiation exposure (7). Colonoscopy has also successfully reversed invaginations in two-thirds of pediatric patients (8) and has also been effective in adult patients (9).

## Future Applications

Laparoscopic augmented reality navigation, LARN, is a novel technique that has shown promise in many surgical procedures, including hepatectomy for liver cancer (10). LARN helps localize important features, like tumors and vessels, and has been shown to reduce intraoperative bleeding, blood transfusion rates, and postoperative recovery time (10). Augmented reality, AR, used with video see-through, gives real-time, three-dimensional anatomical visualization of a variety of structures, including nerves, vessels, lesions, and more. AR has been investigated in neurosurgery (11), otolaryngology (12), urologic laparoscopic surgery (13), and more. AR is more accepted and commonly used in surgeries like these, which are centered around rigid or semi-rigid structures (i.e., bones). Therefore, the use of LARN in those structures that are less rigid and more impacted by respiration, heartbeat, and surgical perturbation has been called into question. Recently, LARN has been successfully applied in a less rigid surgical navigation of the liver (10).

Similar to the liver, the spatial relationships between structures in the digestive tract are inconsistent, and deformations are relatively common. This makes AR-assisted procedures more challenging. Resultantly, there is less data available on the value and efficacy of AR in laparoscopic surgeries of the gastrointestinal tract (14). Nonetheless, the success of the procedure in the stereoscopic laparoscopic hepatectomy of liver cancer in the new methodology from Zhang et al. (10) suggests that these techniques may show promise in gastrointestinal surgeries, like intussusception reductions.

## Conclusion

In the future, the robustness of LARN needs to be further assessed in all surgical procedures, especially digestive organs like the intestines. The following would be required for eventual integration into regular practice for laparoscopic intussusception surgeries: LARN needs to (1) consistently show positive outcomes in the GI tract and (2) enhance enough important information in the visual field of the intestines to outweigh the information that is inevitably occluded (14).

## Author Contributions

All authors listed have made a substantial, direct and intellectual contribution to the work, and approved it for publication.

## Funding

SS and FQ are recipients of the Sarah Morrison Research Award at the University of Missouri–Kansas City School of Medicine.

## Conflict of Interest

The authors declare that the research was conducted in the absence of any commercial or financial relationships that could be construed as a potential conflict of interest.

## Publisher's Note

All claims expressed in this article are solely those of the authors and do not necessarily represent those of their affiliated organizations, or those of the publisher, the editors and the reviewers. Any product that may be evaluated in this article, or claim that may be made by its manufacturer, is not guaranteed or endorsed by the publisher.
